# A Cost-Effectiveness Analysis of Comprehensive Smoking-Cessation Interventions Based on the Community and Hospital Collaboration

**DOI:** 10.3389/fpubh.2022.853438

**Published:** 2022-07-22

**Authors:** Tingting Qin, Qianying Jin, Xingming Li, Xinyuan Bai, Kun Qiao, Mingyu Gu, Yao Wang

**Affiliations:** School of Public Health, Capital Medical University, Beijing, China

**Keywords:** cost-effectiveness, smoking cessation, pharmacological intervention, online health promotion, community health centers

## Abstract

**Background:**

The prevalence of cigarette smoking in China is high and the utilization of smoking cessation clinics is very low. Multicomponent smoking cessation interventions involving community and hospital collaboration have the potential to increase the smoking cessation rate. However, the cost-effectiveness of this intervention model is unknown.

**Methods:**

We conducted a smoking cessation intervention trial in 19 community health service centers in Beijing, China. A cost-effectiveness analysis was performed from a societal perspective to compare three strategies of smoking cessation: no intervention (NI), pharmacological intervention (PI), and comprehensive intervention (CI) (PI plus online health promotion). A Markov model, with a time horizon of 20 years, was used to simulate the natural progression of estimated 10,000 male smokers. A cross-sectional survey was conducted to obtain data on costs and quality-adjusted life years (QALYs) by using the five-level EuroQol-5-dimension (EQ-5D-5L) questionnaire. Probabilistic sensitivity analysis was performed to explore parameters of uncertainty in the model.

**Results:**

A total of 680 participants were included in this study, including 283 in the PI group and 397 in the CI group. After 6 months of follow-up, the smoking cessation rate reached 30.0% in the CI group and 21.2% in the PI group. Using the Markov model, compared with the NI group, the intervention strategies of the PI group and the CI group were found to be cost-effective, with an incremental cost-effectiveness ratio (ICER) of $535.62/QALY and $366.19/QALY, respectively. The probabilistic sensitivity analysis indicated that the CI strategy was always the most cost-effective intervention.

**Conclusion:**

CI for smoking cessation, based in hospital and community in China, is more cost-effective than PI alone. Therefore, this smoking cessation model should be considered to be implemented in healthcare settings.

## Introduction

Tobacco use remains the world's leading preventable cause of morbidity and mortality, resulting in more than 8 million deaths each year worldwide (1). China, the world's largest tobacco producer and consumer, has more than 350 million smokers (2). More than 1 million people die each year from smoking-related diseases, and about 100,000 people die from secondhand smoke exposure in China (3). Worse still, tobacco-related deaths in China are expected to reach 3 million by 2050 if effective measures are not taken to control tobacco use (4). The tobacco epidemic is a major threat to public health in China.

Smoking cessation is one of the most important measures to reduce harm from cigarettes. The Healthy China 2030 Action Plan requires that the prevalence of adult smoking be decreased to 20% by 2030 (5). However, quitting smoking is a complex and difficult process for smokers due to nicotine dependence. The Global Adult Tobacco Survey, Fact Sheet, China, 2018 showed that the smoking rate among people aged 15 and above in China was 26.6%, of which 50.5% of men and 2.1% of women were current smokers (6). Among smokers, only 16.1% of current smokers planned to or were thinking about quitting in the next 12 months, and 90.1% of smokers who tried to quit in the past 12 months did not use any quitting assistance for at least one quit attempt (6). When smokers quit smoking without help, it was difficult to maintain abstinence in long term (7).

In 1998, the World Health Organization (WHO) officially included tobacco dependence as a chronic and highly recurrent disease in the International Classification of Diseases (ICD-10; code F17.2). Therefore, quitting smoking requires scientific and professional guidance, which can strengthen smokers' confidence and determination to quit and help them relieve withdrawal symptoms. Common smoking cessation methods include clinic consultation, pharmacological therapy, nicotine replacement therapy, behavioral intervention, and multi-component smoking cessation treatment. There is ample evidence that the combination of behavioral interventions and pharmacotherapy could improve the effectiveness of smoking cessation (8). In order to meet the demand for smoking cessation services, China has launched a centrally subsidized local smoking cessation clinic project since 2014, which has supported the creation of 586 smoking cessation clinics nationwide (9). However, 57% of the clinics only provide cessation advice and behavioral support, and the utilization rate of these clinics is poor due to a lack of public awareness of their availability (10).

To promote adequate treatment for tobacco dependence, WHO recommends that each country should take appropriate, comprehensive, and integrated measures including establishing population-level and individual level approaches, considering novel approaches and media, and integrating brief advice into the existing primary healthcare system (11). Therefore, it is imperative to offer smoking cessation services in primary healthcare settings, which could achieve better population coverage at a relatively low cost (12). Implementing a smoking cessation program based in the community could mobilize local resources and improve access to smoking cessation services. Several foreign studies have confirmed the feasibility and short-term effectiveness of involving community health workers in smoking cessation (13–15), while there are inadequate studies on long-term effectiveness.

The cost-effectiveness of smoking cessation interventions is increasingly becoming a primary consideration for the public health sector. Some previous literature has been published reporting the cost-effectiveness of different smoking cessation interventions, such as pharmacotherapy, nicotine replacement therapy, and cell phone intervention (15–17). Also, there are various studies using the Markov simulation model to evaluate the cost-effectiveness of tobacco control campaigns in different countries (18–20). To our knowledge, there is little smoking cessation program based on hospital and community collaboration in China. Few studies have demonstrated the cost-effectiveness of this intervention model, which is a good way to examine both the costs and effects of interventions. Therefore, it is imperative to investigate the cost-effectiveness of interventions within the context of China.

This study aims to perform a cost-effectiveness analysis (CEA) of comprehensive smoking-cessation interventions based in the community and hospitals in China to provide economic evidence for the public health program.

## Materials and Methods

### Reporting Guideline

This study is guided by the Consolidated Health Economic Evaluation Reporting Standards (CHEERS) statement (21). The completed CHEERS checklist is provided in Supplementary File 1.

### Design

**Figure 1:**
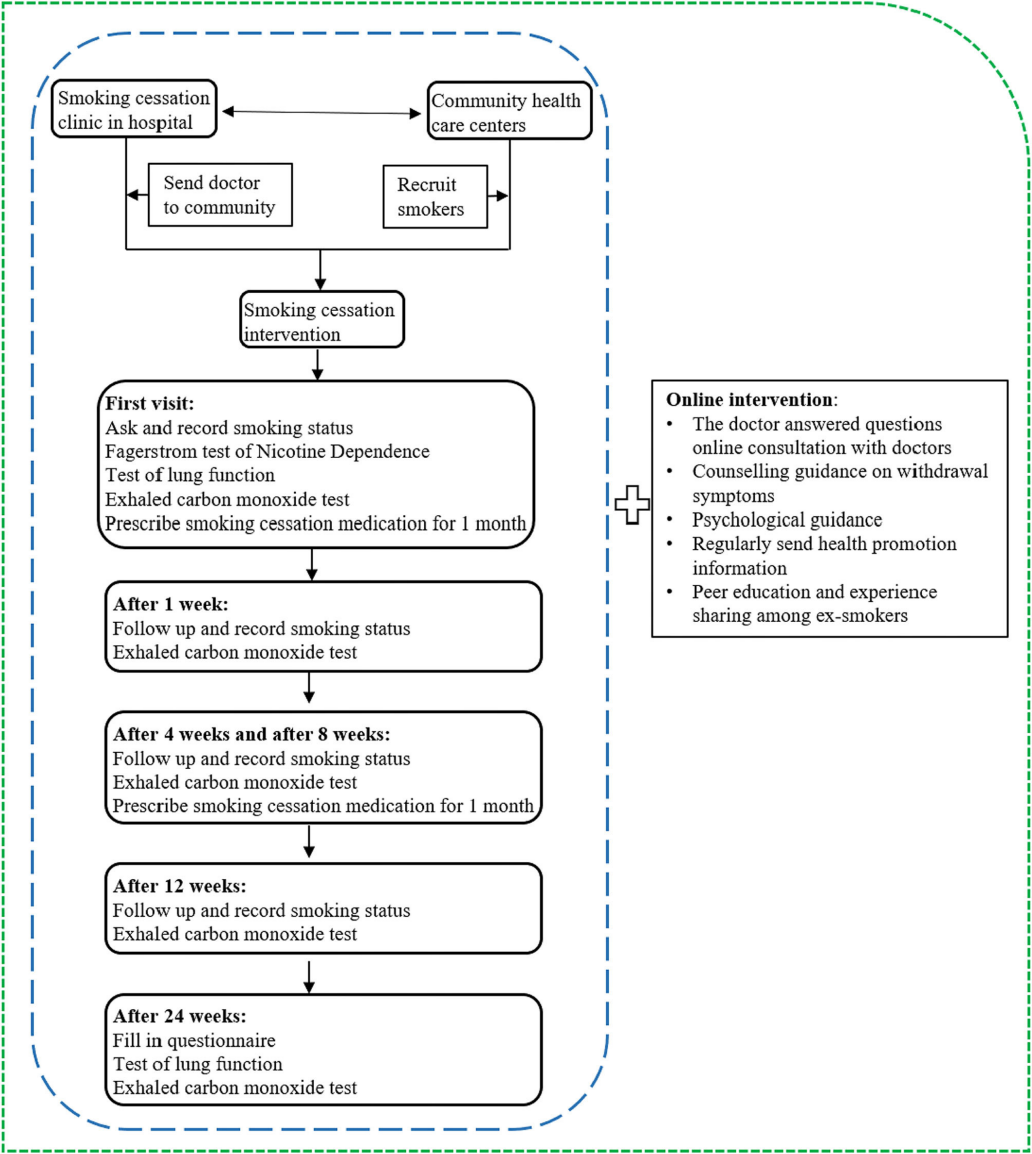
The content of the intervention program. The blue circle and green circle respresent the specific measures of PI and CI, respectively.

This project developed a comprehensive tobacco dependence management model based on community and hospital collaboration and conducted a parallel-controlled community intervention trial to evaluate the effectiveness of the model. The intervention program was implemented by our team at the Capital Medical University, the Respiratory Smoking Cessation Clinic of Sino-Japanese Friendship Hospital, and the community health service centers, which were the intervention sites. Community smoking cessation management groups were formed to be responsible for project implementation. Each group included 20~30 participants, 1 physician from the smoking cessation clinic, 1 community family doctor, 1 community manager, 8~10 volunteers selected from the credible people in the community, and 1 psychological counselor. We compared the cost-effectiveness of three smoking cessation intervention strategies: no intervention (NI), pharmacological intervention (PI), and comprehensive intervention (CI) (PI plus online health promotion). PI and CI were conducted based on community and hospital collaboration. The intervention trial lasted for 6 months, and our team followed up with participants to monitor outcomes such as smoking status, drug use, and smoking cessation willingness. Figure 1 shows the specific measures for each intervention at different time points. For details of the intervention, please see Supplementary File 2.

This study has been approved by the Medical Ethics Committee of Capital Medical University (Z2019SY007). Written informed consent was obtained from all the participants.

### Participants

The sample size for this study was calculated by using the corresponding formula of a two-armed parallel control trial as shown here. The α was 0.05, and the test efficiency was 0.9, which means the β was 0.1. Since the smoking cessation rate of daily smokers over 15 years of age in China was 14.4% according to the 2015 China Adult Tobacco Survey Report, *p*_2_ was 14.4% which reflected the positive rate of the control group. The *p*_1_, the expected positive rate in the test group, was set at 24.4% with the goal of increasing the smoking cessation rate by 10% through intervention. The δ^2^ is the difference between *p*_1_ and *p*_2_, which was 0.10^2^. The required sample size was calculated to be 262. To account for dropout of 5%, we aimed to recruit 276 participants to each group, with a total of 582 participants.


$$\begin{array}{r} n=\frac{{\left(z_{1-\alpha /2}+z_{1-\beta }\right)}^{2}\left[p_{1}\left(1-p_{1}\right)/k+p_{2}\left(1-p_{2}\right)\right]}{\delta^{2}}\\ =\frac{{\left(1.64+1.28\right)}^{2}\times \left[0.144\times 0.856/1+0.244\times 0.756\right]}{0.1^{2}}\approx 262 \end{array}$$

**Figure 2:**
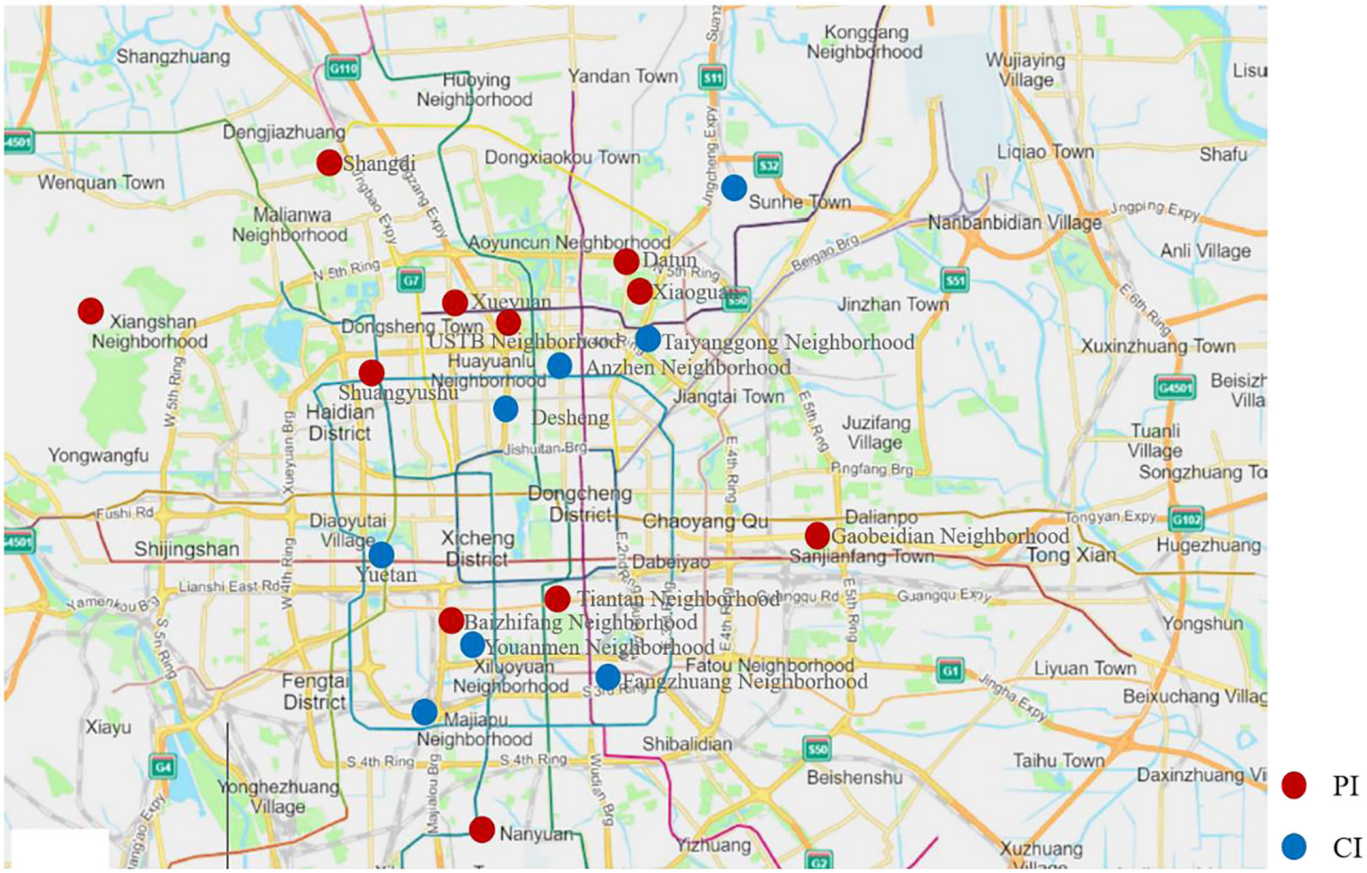
Geographic distribution of sampling communities.

In total, eight community health service centers with a stable population and good basic conditions for chronic disease management were selected as the sites to conduct CI. Matching the characteristics of social economy, population health, and medical services of community health service centers in the CI group, 11 centers were selected as sites to conduct PI. In total, 19 community health service centers in Beijing were selected (Figure 2). Community doctors and community managers were responsible for recruiting smokers who were willing to participate in the smoking cessation intervention program.

Inclusion criteria for the participants were current smokers living in the community who (a) had smoked for 6 months or more in their lifetime and had smoked within 30 days prior to the survey, (b) were not currently using other methods to quit smoking, (c) and could communicate fluently and were willing to take part in the follow-up. Smokers were excluded if they (a) had participated in another smoking cessation program, (b) were pregnant or breast feeding, or (c) were suffering from a serious illness that prevented them from being able to participate physically or mentally.

Prior to the intervention, the investigators were trained by team members. At the intervention site, investigators and doctors from the hospital conducted face-to-face questionnaires, carbon monoxide blow tests, and lung function tests on study participants. Based on these test results, the doctor developed specific smoking cessation plans for each participant, including whether to take smoking cessation drugs, daily doses of the drug, etc. In total, 680 tobacco-dependent smokers were enrolled in our project from 19 communities.

### Decision Model

**Figure 3:**
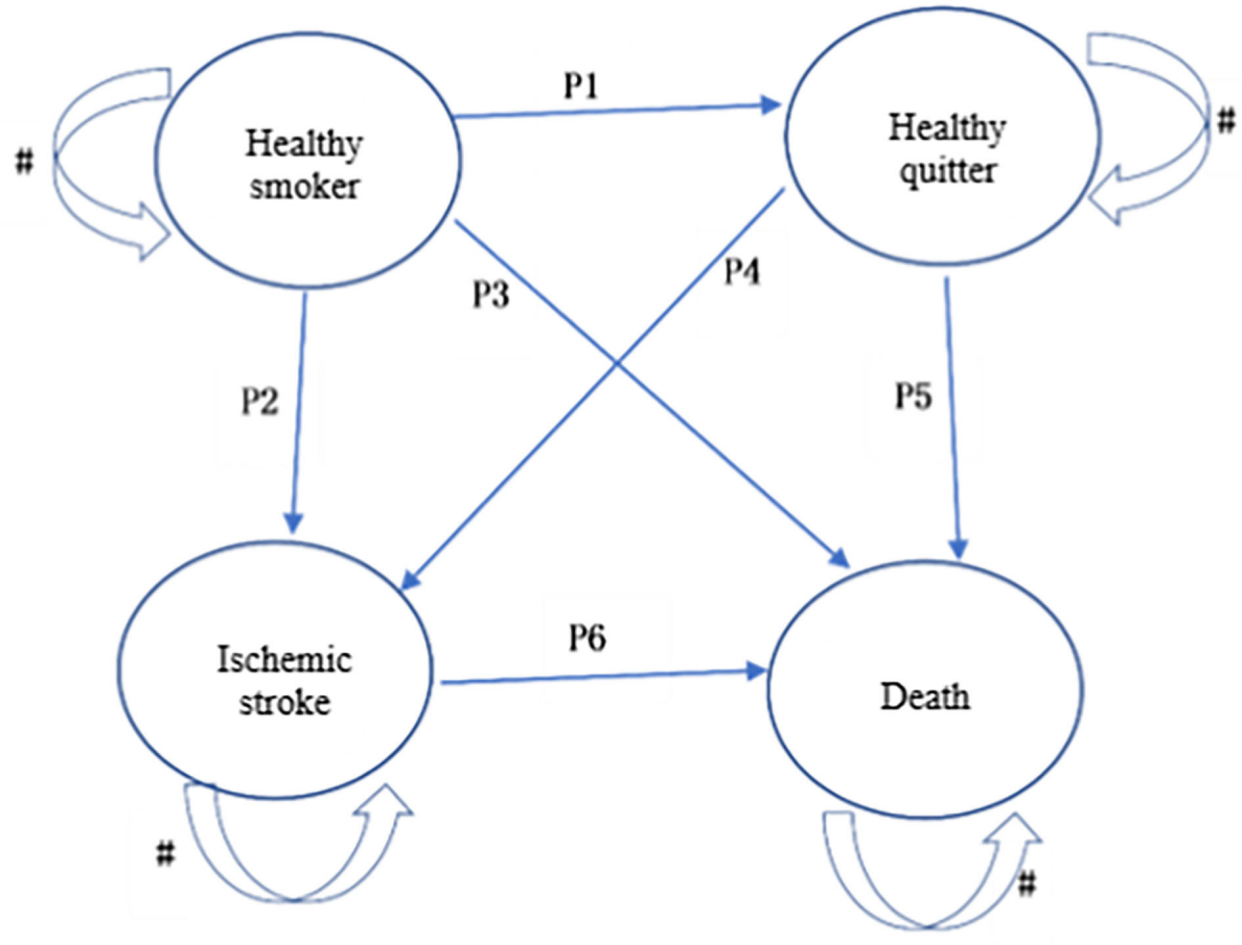
Markov model structure. # indicates that there is a certain probability of remaining within the state.

We created a state-transition Markov model to compare three smoking–cessation intervention strategies by using TreeAge Pro software. Figure 3 illustrates the various states and the potential transitions between these states. The model constructed in this study included four states: healthy smoker, healthy quitter, ischemic stroke, and death. Smoking is a risk factor for a variety of diseases, including cardiovascular disease which is the main smoking-related cause of death globally. In China, the stroke incidence and particularly the incidence of ischemic stroke was higher than in other countries (22). Smoking is a major risk factor for stroke with 10.8% of strokes in men attributed to smoking (23). Thus, in this study, ischemic stroke was selected to represent smoking-related disease and was included in the model.

The base case is 10,000 adult male smokers who were willing to quit smoking. This model, with a time horizon of 20 years, could predict the lifetime costs and quality adjusted life years (QALYs) of different smoking cessation intervention strategies. The model runs on two basic assumptions: (1) a participant can only be in one state in each Markov cycle, and (2) the 'smoker's transition from one state to the next occurs randomly based on probability, regardless of which state the smoker was in before entering the new state. The simulation queue established by this model was 10,000 people with a cycle period of 1 year and a total of 20 cycles. When the time unit of the parameter is inconsistent with the cycle, it is converted to the formula *P*=*(Pt)1/t*, where *P* is the probability of transfer within a cycle. The Markov model framework could be found in Supplementary Figure S1.

### Probabilities

Probabilities data were obtained from the Global Study of the Economic Burden of Disease and the results of domestic epidemiological surveys, and related literature studies are shown in Table 1.

**Table 1:** Decision-analytic model parameters.

**Parameters**	**Estimate**	**Range**	**Reference/source**
**Probabilities**			
RR of ischemic stroke attributed to cigarette smoking	2.32	(1.97–2.67)	(24)
5-year survival rate for ischemic stroke (%)	59.20	(56.4–62.0)	(25)
All-cause mortality (male, 1/100,000)	292.43		(26)
Mortality rate of ischemic stroke (male, 1/100,000)	45.08		(26)
Mortality rate of non-ischemic stroke (male, 1/100,000)	247.35		(26)
Age-standardized incidence rate of ischemic stroke (male, 1/100,000)	266.40		(26)
**Quit rates of difference intervention strategies (%)**			
NI	3.00		(27)
PI	21.20		Intervention trial in this study
CI	30.00		Intervention trial in this study
**Cost (/case)**			
NI group	0.00		
PI group	192.48		Intervention trial in this study
CI group	230.06		Intervention trial in this study
The cost of treatment per capita for ischemic stroke	1361.09		(28)
**Utility**			
Utility of smoker	0.96	(0.88–1.00)	Intervention trial in this study
Utility of quitter	1.00		
Utility of patients with ischemic stroke	0.76	(0.69–0.82)	(28, 29)
**Other**			
China GDP per capita for 2019	10279.12		(30)

### Costs

The costs of different smoking cessation intervention strategies were calculated using the work decomposition structure (WBS) and the operation cost method to count the input of each group of resources in the community intervention trial. The average cost of hospitalization for ischemic stroke comes from the *China Stroke Prevention and Control Report 2019*. The cost estimates in this study are divided into average direct costs and average indirect costs. The average direct costs include inspection costs, smoking cessation drug costs, and other material costs. The average indirect costs include medical “personnel's lost work expense,” smoker's lost work expense, and transportation cost. The total costs of the different smoking cessation strategies are shown in Table 1. All the costs were inflated to 2019 using the China Consumer Price Index and converted to US dollars using official exchange rates as of 2019 (US$1 = ¥6.90).

### Quality-Adjusted Life Years (QALYs)

Quality-adjusted life years as a measurement reflecting both health-related quality of life and mortality is recommended by China Guidelines for Pharmacoeconomic Evaluations as the most suitable measure for the economic evaluation of health outcomes. QALYs are equal to time spent in the relevant health states multiplied by an appropriate utility score. In this study, a cross-sectional survey was conducted to identify the utility scores for smokers, in which participants completed EuroQol five-dimensional questionnaires (EQ-5D-5L). The EQ-5D-5L utility scores are shown in Table 1.

### Analysis

We conducted our analysis from a societal perspective in accordance with the China Guidelines for Pharmacoeconomic Evaluations. A hypothetical cohort of base case adult smokers was simulated over a 20-year time horizon with each cycle lasting 1 year. We discounted future costs and future benefits at 3%. Outcome measures were reported in incremental cost-effectiveness ratios (ICERs) with a Cost-Effectiveness Threshold (CET) of 1–3 times GDP per capita in China (10,279.12 ~ 30837.36) (30), which was the most common approach to set CET (31). The intervention strategy would be considered to be “very” cost-effective if ICER was less than GDP per capita, and could still count as cost-effective if the ICER did not exceed 3 times of GDP per capita (32). By using the probabilistic sensitivity analyses, we explored the uncertainty of model parameters, including incidence rate of ischemic stroke, and utility values. A Cost-effectiveness acceptability curve was generated to illustrate the percentage of curves estimated by ICER that were cost-effective at different values of willingness to pay per quit.

## Results

Of all the 680 smokers who participated across communities, 283 were randomly assigned to the PI group and 397 to the CI group (Table 2). In the PI group, there were 251 male smokers (88.7%), and most participants had a secondary education level and above (89.7%). In the CI group, 366 participants (92.2%) were male, and about 193 smokers (49.0%) had retired. About two-thirds of participants had at least moderate FTND defined nicotine dependence in both the PI group (62.6%) and CI group (62.9%). Baseline characteristics between the two groups were similar.

**Table 2:** Baseline characteristics of participants.

**Characteristics**	**PI group (*n* = 283), *N* (%)**	**CI group (*n* = 397), *N* (%)**
**Sex**		
Male	251 (88.7)	366 (92.2)
Female	32 (11.3)	31 (7.8)
**Age**		
<40	55 (19.5)	50 (12.6)
40~49	51 (18.1)	50 (12.6)
50~59	84 (30.0)	121 (30.5)
≥60	91 (32.4)	176 (44.3)
**Marital status**		
Unmarried	21 (7.4)	17 (4.3)
Married	239 (84.8)	356 (89.9)
Other	22 (7.8)	23 (5.8)
**Education level**		
Primary or lower	29 (10.3)	17 (4.3)
Secondary	145 (51.4)	219 (55.2)
Tertiary	108 (38.3)	161 (40.6)
**Employment**		
Production operations clerks	33 (11.8)	38 (9.6)
Business services personnel	45 (16.1)	34 (8.6)
Staff of state organizations and enterprises	25 (8.9)	44 (11.2)
Professional and technical personnel	28 (10.0)	30 (7.6)
Military, students and other unemployed workers	52 (18.6)	55 (14.0)
Retirees	97 (34.6)	193 (49.0)
**Personal monthly income (Chinese Yuan)**		
≤ 2,000	31 (12.7)	46 (13.0)
2,001~4,000	79 (32.2)	113 (31.9)
4,001~6,000	53 (21.6)	104 (29.4)
6,001~8,000	31 (12.7)	36 (10.2)
8,001~10,000	23 (9.4)	23 (6.5)
>10,000	28 (11.4)	32 (9.0)
FTND^*^		
Mild	95 (37.4)	133 (37.2)
Moderate	101 (39.8)	152 (42.5)
Severe	58 (22.8)	73 (20.4)

* *Fagerstrom Test for Nicotine Dependence*.

Table 3 shows the short-term effects in the CI and PI groups after receiving the smoking cessation intervention. After 1 month, 54 participants in the CI group and 26 in the PI group successfully quit smoking, and the difference between the two groups was not statistically significant (*p* > 0.05). After 3 months of the intervention, 81 people in the CI group and 38 people in the PI group successfully quit smoking, and the results showed a statistically significant difference in the distribution of intervention effect between the two groups (*p* < 0.05). After 6 months, the smoking cessation rate of the CI and PI groups was 30.0 and 21.2%, respectively (*p* < 0.05).

**Table 3:** Short-term effects in the intervention groups after receiving smoking cessation intervention.

**Smoking status**	**CI group**	**PI group**	***x^**2**^***	***p***
**After 1 month**				
Quit smoking	54 (13.6)	26 (9.2)	3.102	0.078
Continue smoking	343 (86.4)	257 (90.8)		
**After 3 months**				
Quit smoking	81 (20.4)	38 (13.4)	5.568	0.018
Continue smoking	316 (79.6)	245 (86.6)		
**After 6 months**				
Quit smoking	119 (30.0)	60 (21.2)	6.557	0.010
Continue smoking	278 (70.0)	223 (78.8)		

Table 4 shows the cost, QALYs, incremental costs, and the ICER of different intervention strategies. Compared with the NI group, the intervention strategy of the PI group and the CI group were both found to be very cost-effective with ICER of $535.62/QALY and $366.19/QALY, respectively, which were well below our CET ($10279.12/QALY).

**Table 4:** Modeled cost-effectiveness ratios based on 10,000 people.

**Intervention strategies**	**Cost**	**QALYs**	**Cost per capita**	**QALYs per capita**	**Incremental cost per capita**	**Incremental QALYs per capita**	**ICER**
NI	1739813.56	195,034	173.98	19.503	–	–	–
PI	3372396.81	198,082	337.24	19.808	163.26	0.305	535.62
CI	3550971.10	199,930	355.10	19.993	181.12	0.490	366.19

### Sensitivity Analysis

**Figure 4:**
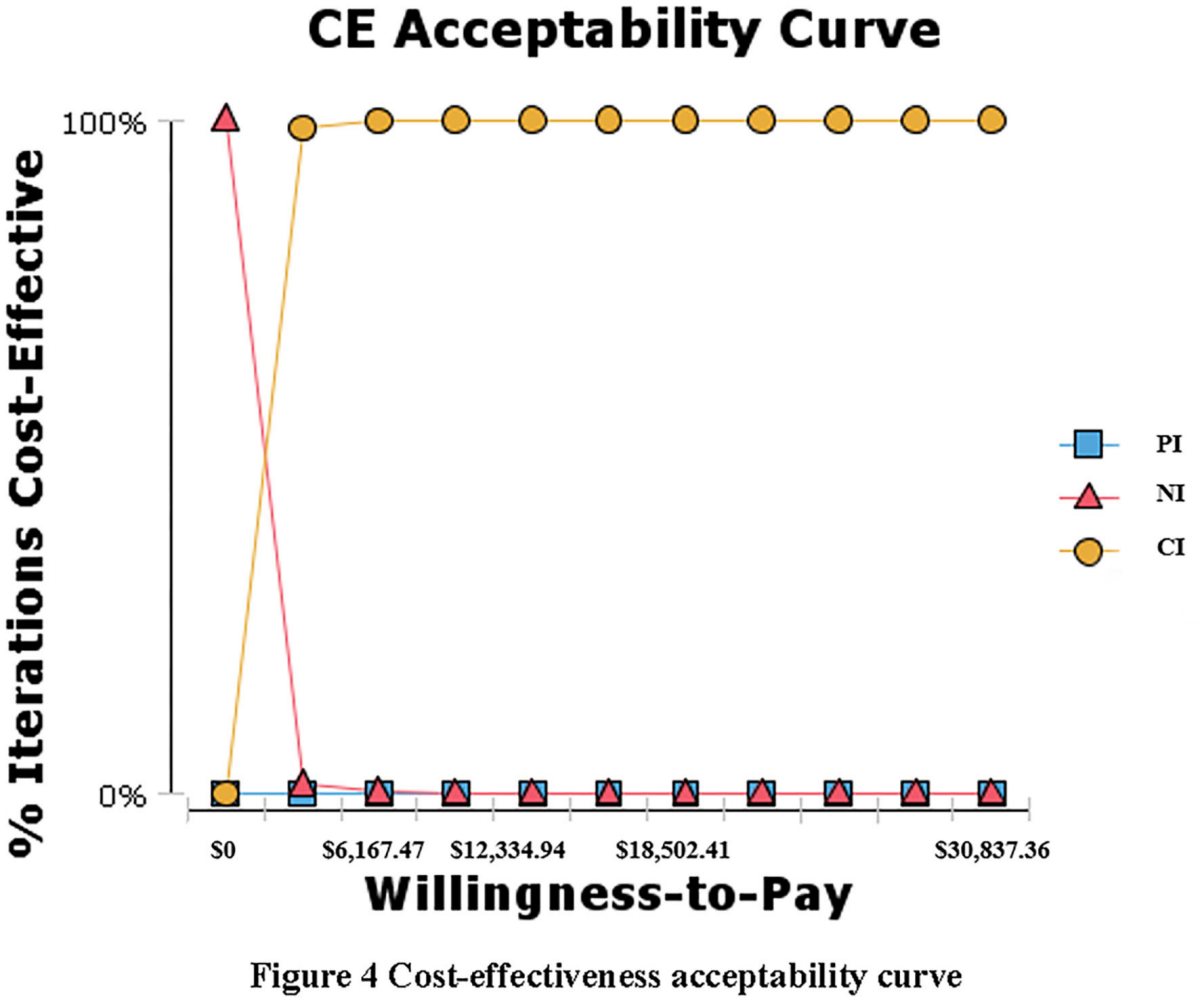
Cost-effectiveness acceptability curve.

In this study, the probability sensitivity of incidence rate and utility indicators including age-standardized incidence rate of ischemic stroke, the utility of smoker, quitter, and patients with ischemic stroke, respectively, in Table 1 were analyzed. Sensitivity analysis results suggest that when the willingness to pay threshold was >$0/QALY, the CI strategy was always the most cost-effective intervention plan, while the PI strategy was considered to be more costly and less effective (Figure 4).

## Discussion

To our knowledge, this study is the first to compare the relative costs and outcomes of different smoking cessation intervention strategies based on community and hospital collaboration, in China. Our findings indicate that compared with NI, the CI program based on hospital and community collaboration is cost-effective. Through sensitivity analysis simulating the effects of adjusting for a range of bias parameters, we found that our model has good stability statistically. These findings should inform the formulation of effective tobacco control policies in China.

In 2013, the WHO proposed that primary healthcare should play an important role in tobacco control (33). According to WHO estimates, the possible effects of integrating smoking cessation interventions for all smokers into basic healthcare services include: more than 80% of all smokers would be served each year, 40% of smokers would be encouraged to try to quit, and 2~3% of smokers would quit smoking successfully. This indicates that providing smoking cessation services in primary healthcare is effective for tobacco control. In the UK, smoking cessation interventions and clinical treatment referrals for smoking cessation by community GPs were introduced for specific groups, in 2002 (34). In the United States, Australia, and other countries, community-oriented tobacco control interventions have also been carried out for many years (13, 35, 36). Unlike foreign countries that rely on community pharmacy personnel to provide smoking cessation services (37), family doctors and physicians from smoking cessation clinics were responsible to provide services in this study, due to the family doctor contract services (FDCSs) in China. Based on institutional characteristics in China, this approach could not only save costs but also improve the effects of smoking cessation. In recent years, the chronic disease management model of a hospital–community linkage has been applied to the intervention management of many chronic diseases and has achieved good management results (38). However, community health service centers, as the provider of primary healthcare services, have delivered little in terms of services for tobacco control in China.

This study performed a cost-effectiveness analysis alongside a randomized controlled quasi-experiment involving the community and hospital. From the societal perspective, our findings suggest that the CI program based on community and hospital collaboration is cost saving, which means that the cost of the intervention program is lower compared with the cost of smoking-related diseases. This indicates that health policymakers should consider putting part of the budget allocations for tobacco control into community healthcare centers. In addition, this study confirmed the significance of online health promotion intervention in smoking cessation, which is consistent with previous findings (39). Paying attention to the smokers' quitting needs and obstacles they encountered at different stages and providing the social support they need is critical to increasing the smoking cessation rate (40). Online health promotion services can provide smokers with the information and emotional support they need timely and conveniently, and thus, may be considered a great way to assist smoking cessation.

Our findings indicate that the smoking cessation model based on hospital and community collaboration may be feasible and acceptable for tobacco control. In view of the prevalence of tobacco use, in order to better control smoking, it is recommended to promote hospital-community multisectoral cooperation with the tobacco dependence management model. First, we should establish a multi-sectoral cooperative smoking cessation support system with the community as the place and the hospital as the support system. Training is required to strengthen the knowledge and skills of relevant community medical personnel. Also, we should establish shared online resources containing relevant health promotion materials by using the Internet, WeChat group, which is one of the most popular social media in China, and other new media. This resource would support follow-up management, enrich the community smoking cessation intervention system, and strengthen the implementation of relevant policies to promote the sustainable development of hospital–community smoking cessation interventions.

### Limitations

Several limitations of our study should be carefully considered. First, in the community intervention trial, the investigation of re-smoking after smoking cessation was not studied, so the effect of smoking cessation and relapse on the smoking cessation population was not considered within the model. This may have magnified the effect of quitting smoking, resulting in a certain degree of bias. In addition, this study only selected ischemic stroke to predict smoking-related diseases in the model, and only accounted for the direct treatment cost of ischemic stroke. Smoking is a strong risk factor for many diseases. Therefore, other diseases should also be considered in states prediction for further studies and simulations. The model in this study is based on adult smoking men, and the model prediction is not specific to different age groups. Since there may be some differences in the effects and long-term benefits of smoking cessation in different age groups, further research is required to investigate this.

### Summary

In summary, the CI for smoking cessation based on hospital and community collaboration in China has been shown to be very cost-effective. This smoking cessation model should be further implemented and evaluated to more strongly establish the potential benefit to public health in China.

## Clinical Trial Registration Number

This study has been registered on the official website of the China Clinical Trial Registration Centre (ChiCTR1900024991).

## Data Availability Statement

The datasets used and/or analyzed during the current study are available from the corresponding author on reasonable request.

## Ethics Statement

The studies involving human participants were reviewed and approved by the Medical Ethics Committee of Capital Medical University. The patients/participants provided their written informed consent to participate in this study.

## Author Contributions

TQ: conceptualization, methodology, formal analysis, writing—original draft, and writing—review and editing. QJ: conceptualization, methodology, data curation, and formal analysis. XL: conceptualization, methodology, investigation, resources, supervision, project administration, and writing—review and editing. XB: investigation, project administration, and data acquisition. KQ: conceptualization, investigation, and data curation. MG: investigation and writing—review and editing. YW: investigation, resources, and project administration. All authors contributed to the article and approved the submitted version.

## Funding

This research was supported by the Foundation of the National Key R&D Program of China (2017YFC1309404).

## Conflict of Interest

The authors declare that the research was conducted in the absence of any commercial or financial relationships that could be construed as a potential conflict of interest.

## Publisher's Note

All claims expressed in this article are solely those of the authors and do not necessarily represent those of their affiliated organizations, or those of the publisher, the editors and the reviewers. Any product that may be evaluated in this article, or claim that may be made by its manufacturer, is not guaranteed or endorsed by the publisher.
