# Supplementary material for: A Cost-Effectiveness Analysis of Comprehensive Smoking-Cessation Interventions Based on the Community and Hospital Collaboration
Source: Front Public Health. 2022 Jul 22;10:853438. doi: 10.3389/fpubh.2022.853438 (PMC9354545; doi:10.3389/fpubh.2022.853438)
Supplement: Supplementary file 1 [file Data_Sheet_1.pdf]

Supplementary Material

Study protocol

On the basis of previous expert consultation and on-site interviews, a hospital-community comprehensive tobacco dependence management model was initially formed. During the development of this model, the research group had built a consensus with multiple institutions including the Respiratory Smoking Cessation Clinic of Sino-Japanese Friendship Hospital, community health service centers, health education centers, community management institutions and Beijing Tobacco Control Association. Each institution sent a special person to jointly form the service management team of this project, and their respective responsibilities was clarified (Table s1).

Table S2 The responsibility of each institution participating in the project

Institutions	Specific tasks of the project implementation			
	**Community promotion and mobilization**	**Screening and information connection**	**Documentation and participants classification**	**Intervention and follow-up management**
the Respiratory Smoking Cessation Clinic of Sino-Japanese Friendship Hospital	Cooperate with the development of health promotion and health education materials	Physical examination of participants (including pulmonary function tests)	Participate in tobacco dependence assessment and drug use assessment	Tobacco-related health counseling, medication and health coaching
community health service centers	Cooperate with the research group in community mobilization and recruitment of participants	Provide the resident registration information to the research group	Provide activity settings and participate in blood draws for participants	Participate in follow-up, counseling and health education
community management institutions	Community mobilization and	Information connection with community	Coordinate community health centers and general hospitals	Continuous tobacco control health education and community smoke-free

Institutions	Specific tasks of the project implementation			
	**Community promotion and mobilization**	**Screening and information connection**	**Documentation and participants classification**	**Intervention and follow-up management**
	participants recruitment	health centers and research group		environment construction
Beijing Tobacco Control Association	Organize and coordinate management agencies such as related streets and neighborhood committees	Organize and coordinate and connect with community health centers information	Organize and connect with community health centers information	Participate in community volunteer smoking cessation training
health education centers	Responsible for the design and development of health education materials	Provide health education information	Connection of health education materials and information network platform	Create smoking cessation environment and provide health education and publicity materials
research group	project design, community organization and participants recruitment	Process supervision, and management of enrolled participants	Organize information collection and management of participants	Monitor the compliance, and evaluate intervention effects

Based on the content of intervention program in Fig.1, we have added more specific details to illustrate the study process as the followings:

The community health service centers were the main place for intervention implementation. Participants with smoking dependence could access comprehensive and continuous intervention services to quit smoking without leaving the community. Based on the advantages of family doctor contract services (FDCS), the community health service centers were responsible for mobilizing and recruiting smokers, assisting the intervention team in selecting and training volunteers, providing places for regular activities, and assisting volunteers and doctors in carrying out follow-up management and other work. The activities carried out in the community were mainly divided into

four parts: recruitment of participants, information collection and evaluation, implementation of intervention, effect evaluation and feedback.

(1) Recruitment of participants: The community mobilized the family doctor service teams of FDCS to ask about the smoking history of residents, and encourage them to participate in community smoking cessation intervention activities based on a preliminary assessment of their willingness to quit smoking.

(2) Information collection and evaluation: The collected information included ① baseline information covered demographic information, smoking history, smoking cessation attempts, awareness of the harms of smoking, intention to quitting smoking, and psychological assessment; ② exhaled carbon monoxide test; ③ lung function test; ④ blood biochemical test. On the basis of these above information, doctors from smoking cessation clinic assessed the smokers' degree of tobacco dependence, psychological status, personal smoking cessation needs, reasons for the failure of smoking cessation, and randomly classified them into pharmacological intervention (PI) group or comprehensive intervention (CI) (PI plus online health promotion) group. The baseline survey took each participant about 40-50 minutes to finish, while the follow up took about 20~30 minutes.

(3) Implementation of intervention: The PI group mainly received pharmacological intervention, while the CI group received both PI and online health promotion intervention (Table S2). Pharmacological intervention was mainly carried out in community health service centers, where physicians from the smoking cessation clinic provided individualized medication guidance based on participants' smoking status. The main drug used in this project was varenicline tartrate tablets. Online health promotion intervention was mainly conducted through WeChat groups. WeChat, a social media for muti-purpose instant messaging and calling, has become one of the main ways people communicate in China. Based on the WeChat groups of participants in different communities, health education centers regularly sent education information weekly, physicians from smoking cessation clinic and family doctors answered questions online, and psychological professionals provided counseling services for smokers who had withdrawal symptoms. In addition, we could notify the follow-up matters in the WeChat groups, and participants could also discuss online to share their smoking cessation feelings and experiences, which may be helpful for them to successfully quit smoking. The online health education materials, designed based on the cognitive behavioral theory and the theory of "knowing, believing and doing", varied according to the time point, including a set of electronic documents and micro-videos. These materials provided for participants aimed to enhance the motivation and confidence of smoking cessation and help participants to get rid of nicotine dependence effectively.

Table S3 Detailed interventions for two groups

Group	Interventions
PI group	**Pharmacological intervention:**<br><br>In community health centers, physicians provided participants with individualized medication guidance at the first visit, one month later, and two months later based on their smoking status.

Group	Interventions
CI group	**Pharmacological intervention:**<br>Same as the above.<br>**Online health promotion intervention:**<br>Professionals from health education centers sent education information to multiple WeChat groups weekly.<br>Physicians from smoking cessation clinic and family doctors answered questions raised by participants through WeChat groups.<br>Psychological professionals provided counseling services for smokers who had withdrawal symptoms.<br>Participants could discuss online to share their smoking cessation feelings and experiences.

(4) Follow-up: In this study, we provided participants with three-month smoking cessation intervention services and six-month follow-up after enrollment. We followed up at 1, 4, 8, and 12 weeks after the intervention, and a final survey was finished at 24 weeks after the intervention. To complete the follow-up, face-to-face or telephone interview was carried out due to the impact of coronavirus pandemic. The content of follow-up mainly includes asking and recording smoking status, testing carbon monoxide metabolism level and providing guidance of smoking cessation drugs in the next stage.

(5) Effect evaluation and feedback: Participants were asked about their smoking status during each follow-up period by face to face or telephone questionnaire survey, which included whether to quit smoking, daily cigarette consumption, etc. A final survey was conducted 6 months after the intervention. And the smoking cessation effect information was fed back to participants to enhance their self-confidence in quitting smoking.
