# Supplementary figures and images for: A Cost-Effectiveness Analysis of Comprehensive Smoking-Cessation Interventions Based on the Community and Hospital Collaboration
Source: Front Public Health. 2022 Jul 22;10:853438. doi: 10.3389/fpubh.2022.853438 (PMC9354545; doi:10.3389/fpubh.2022.853438)

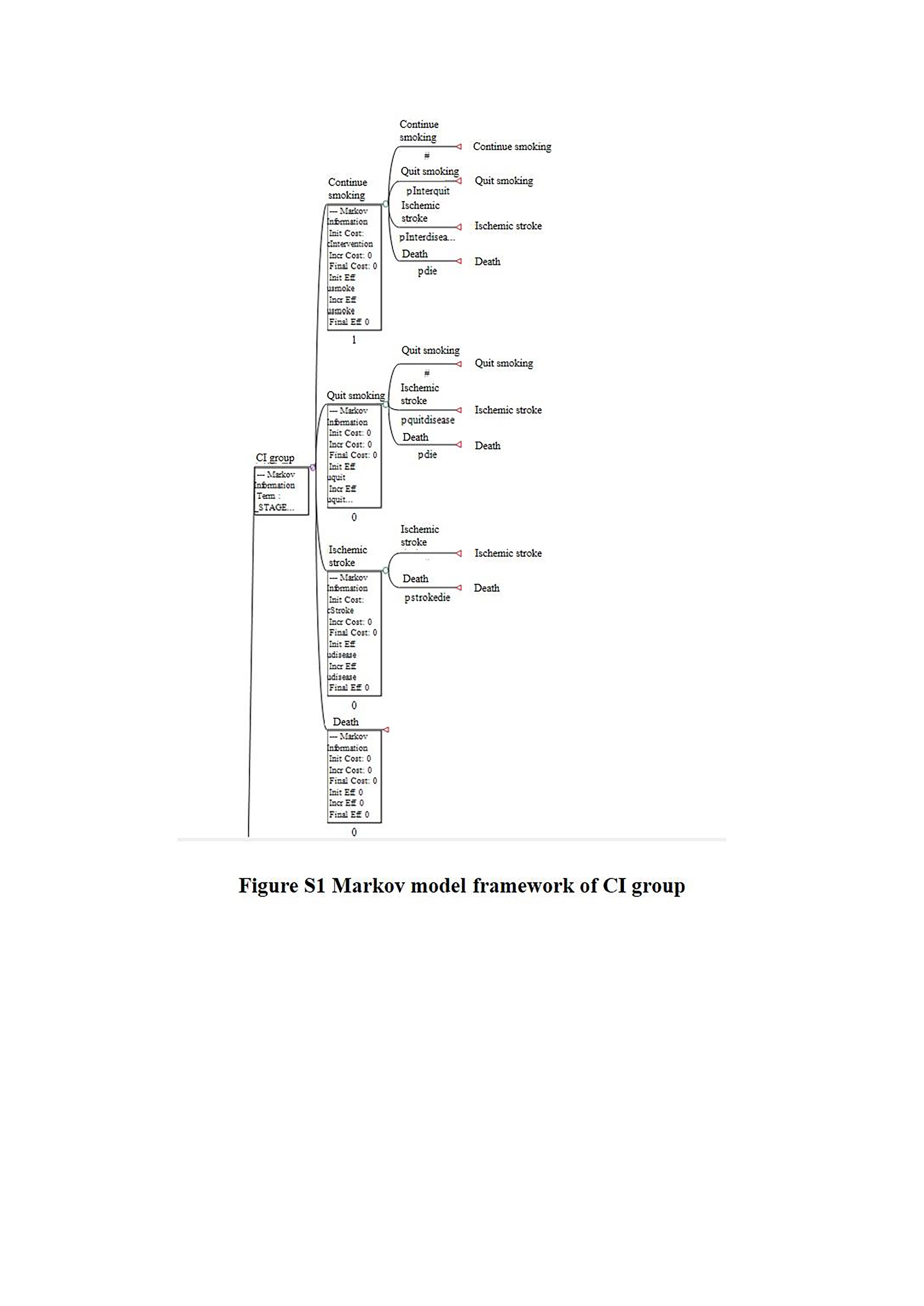

Supplement: Supplementary file 3 [file Figure_1.TIF]
